# Quantitative mass spectrometry for human melanocortin peptides *in vitro* and *in vivo* suggests prominent roles for β-MSH and desacetyl α-MSH in energy homeostasis

**DOI:** 10.1016/j.molmet.2018.08.006

**Published:** 2018-08-21

**Authors:** Peter Kirwan, Richard G. Kay, Bas Brouwers, Vicente Herranz-Pérez, Magdalena Jura, Pierre Larraufie, Julie Jerber, Jason Pembroke, Theresa Bartels, Anne White, Fiona M. Gribble, Frank Reimann, I. Sadaf Farooqi, Stephen O'Rahilly, Florian T. Merkle

**Affiliations:** 1 Metabolic Research Laboratories and Medical Research Council Metabolic Diseases Unit, Wellcome Trust-Medical Research Council Institute of Metabolic Science, University of Cambridge, Cambridge, CB2 0QQ, UK; 2 The Anne McLaren Laboratory for Regenerative Medicine, Wellcome Trust-Medical Research Council Cambridge Stem Cell Institute, University of Cambridge, Cambridge, CB2 0SZ, UK; 3 Laboratory of Comparative Neurobiology, Cavanilles Institute of Biodiversity and Evolutionary Biology, University of Valencia, CIBERNED, 46980 Valencia, Spain; 4 Predepartamental Unit of Medicine, Faculty of Health Sciences, Universitat Jaume I, 12071 Castelló de la Plana, Spain; 5 Open Targets, Wellcome Trust Sanger Institute, Hinxton, CB10 1SA, UK; 6 LGC Ltd., Newmarket Road, Fordham, Cambridgeshire, CB7 5WW, UK; 7 Division of Diabetes, Endocrinology and Gastroenterology, Faculty of Biology, Medicine and Health, University of Manchester, Manchester, UK

**Keywords:** POMC, Human pluripotent stem cell, Obesity, MSH, Leptin, Neuropeptide, hPSC, human pluripotent stem cells, LC-MS/MS, liquid chromatography tandem mass spectrometry, PVH, the paraventricular nucleus of the hypothalamus, CTX, cerebral cortex

## Abstract

**Objective:**

The lack of pro-opiomelanocortin (POMC)-derived melanocortin peptides results in hypoadrenalism and severe obesity in both humans and rodents that is treatable with synthetic melanocortins. However, there are significant differences in POMC processing between humans and rodents, and little is known about the relative physiological importance of POMC products in the human brain. The aim of this study was to determine which POMC-derived peptides are present in the human brain, to establish their relative concentrations, and to test if their production is dynamically regulated.

**Methods:**

We analysed both fresh post-mortem human hypothalamic tissue and hypothalamic neurons derived from human pluripotent stem cells (hPSCs) using liquid chromatography tandem mass spectrometry (LC-MS/MS) to determine the sequence and quantify the production of hypothalamic neuropeptides, including those derived from POMC.

**Results:**

In both *in vitro* and *in vivo* hypothalamic cells, LC-MS/MS revealed the sequence of hundreds of neuropeptides as a resource for the field. Although the existence of β-melanocyte stimulating hormone (MSH) is controversial, we found that both this peptide and desacetyl α-MSH (d-α-MSH) were produced in considerable excess of acetylated α-MSH. In hPSC-derived hypothalamic neurons, these POMC derivatives were appropriately trafficked, secreted, and their production was significantly (P < 0.0001) increased in response to the hormone leptin.

**Conclusions:**

Our findings challenge the assumed pre-eminence of α-MSH and suggest that in humans, d-α-MSH and β-MSH are likely to be the predominant physiological products acting on melanocortin receptors.

## Highlights

• Characterisation of the human hypothalamic peptidome *in vitro* and *in vivo*.
• hPSC-derived POMC neurons correctly process POMC into peptides important for regulating feeding and energy balance, including β-MSH.
• β-MSH and desacetyl α-MSH are produced in excess of acetylated α-MSH in primary and hPSC-derived human hypothalamic cells.
• Leptin significantly increases the production of human POMC-derived peptides.

## 1 Introduction

In order to develop effective treatments for obesity, it is important to understand the mechanisms that regulate energy homeostasis. Hypothalamic neurons that produce pro-opiomelanocortin (POMC) are important regulators of energy homeostasis in both mice [1] and humans [2]. Severely obese patients with POMC deficiency dramatically lose weight in response to a drug mimicking the effects of POMC-derived peptides [3]. The potential of these drugs to find more widespread clinical use highlights the importance of better understanding human POMC biology.

**Figure 1:**
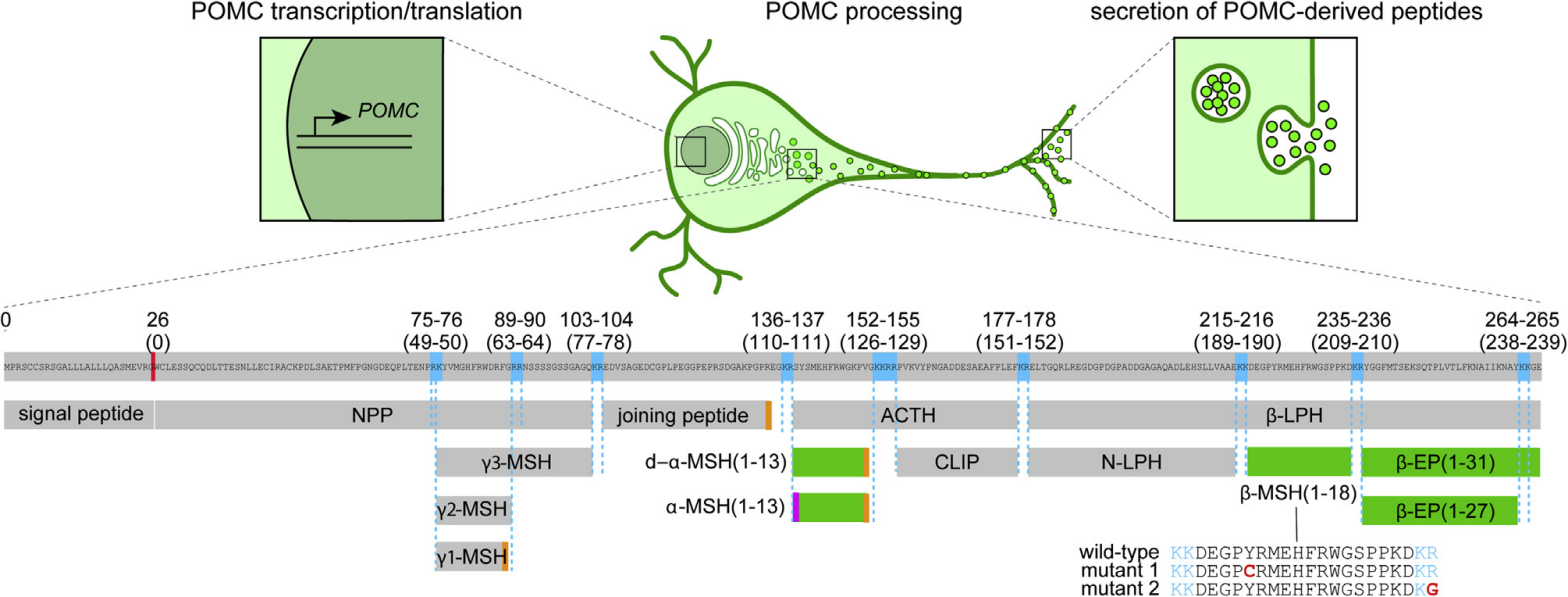
Regulation of POMC processing and secretion. Human POMC is translated as a 267-amino acid protein that, after removal of the signal peptide, undergoes successive rounds of cleavage and trimming at dibasic residues (blue) in a tissue-specific manner; the hypothalamic pattern is illustrated. Additional levels of post-translational modification include C-terminal amidation (orange) and N-terminal acetylation (magenta). The most extensively characterised POMC-derived peptides that regulate food intake (green) include d-α-MSH(1-13), α-MSH(1-13), β-MSH(1-18), β-EP (1-31) and β-EP (1-27). Illustrated mutations in β-MSH have been associated with obesity, suggesting a role for this peptide in human body weight regulation. The concentrations of secreted POMC-derived peptides may be regulated at the levels of transcription, translation, processing, and secretion. ACTH, adrenocorticotropic hormone; CLIP, corticotropin-like intermediary peptide; EP, endorphin; LPH, lipotropin; MSH, melanocyte stimulating hormone; NPP, POMC N-terminal region; POMC, pro-opiomelanocortin.

POMC protein undergoes extensive proteolytic cleavage to produce neuropeptides that regulate food intake and energy expenditure [4], [5] (Figure 1). These peptides include desacetyl α-melanocyte stimulating hormone (ACTH(1-13) amide, d-α-MSH(1-13), d-α-MSH), which can also be acetylated to produce α-MSH(1-13). Both d-α-MSH and α-MSH stimulate melanocortin receptor 4 (MC4R) in the brain to reduce food intake [6], [7], [8], [9]. POMC is also processed into β-endorphin (β-EP (1-31)) [10], [11] which stimulates the μ-opioid receptor to promote analgesia and increase food intake [12], [13], [14]. Human hypothalamic POMC neurons might also produce β-MSH [15], which is associated with human obesity when mutated [16], [17], [18] and inhibits feeding as potently as α-MSH when injected into mice [18] by activating MC4R [17], [18], [19]. However, β-MSH is not produced in the rodent brain due to coding variants at the N-terminus of β-MSH that disrupt the dibasic cleavage site conserved in many other vertebrates, and its existence and biological relevance in humans remains controversial [20], [21], [22].

Since body weight is sensitive to changes in the concentration of POMC-derived peptides [23], [24], [25], [26], [27], understanding POMC processing can provide insights into molecular mechanisms of obesity that could be therapeutically harnessed [4]. Since POMC processing is incomplete even in wild-type brains [10], [11], stimulating POMC production or promoting its processing might reduce body weight in obesity. For example, the adipocyte-derived hormone leptin promotes POMC gene expression [26], [28] and POMC processing [29], [30] to increase α-MSH production in mice.

While studies in mice have been valuable, their inability to produce β-MSH limits their utility as a model system for studying human-specific aspects of POMC processing. Post-mortem human brain samples provide a snapshot of peptide production, but cannot provide information on the dynamic regulation of POMC processing and secretion. To address these issues, we [31], [32] and others [33], [34], [35] developed methods to generate hypothalamic neurons in culture from human pluripotent stem cells (hPSCs). Since these hypothalamic neurons can be produced at scale and are readily manipulated, they provide an unprecedented opportunity to study human POMC biology. POMC-derived peptides are typically quantified using ELISAs, which provide information about target peptides but cannot discriminate between peptides that share common epitopes but that might have functionally-relevant structural differences [11], [36], [37], [38]. In contrast, liquid chromatography tandem mass spectrometry (LC-MS/MS) is sensitive and highly specific, providing information on the precise sequence and post-translational modifications of thousands of peptides in a single experiment [39].

Here, we used LC-MS/MS to analyse POMC processing in both hPSC-derived hypothalamic neurons and primary human brain samples to address several outstanding questions: *Is β-MSH produced in the human brain? What are the relative concentrations of POMC-derived peptides that regulate human energy homeostasis? Are the concentrations of these peptides regulated by leptin in humans*, *as they are in mice?*

We found that hPSC-derived hypothalamic neurons trafficked and secreted POMC and its derivatives, including β-MSH(1-18), and appropriately processed POMC into neuropeptides previously established to regulate energy balance. We next developed quantitative assays for POMC-derived peptides and found that acetylated α-MSH was present at substantially lower concentrations than d-α-MSH, β-MSH, or β-EP both *in vitro* and *in vivo*. Treatment with the adipocyte-derived hormone leptin significantly promoted the production of these peptides in hPSC-derived hypothalamic neurons, establishing them as a valuable tool for studying the dynamics of POMC processing and secretion. Overall, these findings suggest that the importance of β-MSH and d-α-MSH in human obesity has been under-appreciated.

## 2 Materials and methods

### 2.1 Differentiation of hPSCs to hypothalamic and cortical neurons

Pluripotent stem cell maintenance, hypothalamic differentiation [31], [32], and cortical differentiation [40] were carried out as previously described. Cell lines used were WA09 (H9) hESCs (Passage 38-42, WiCell, RRID: CVCL_9773), HUES9 hESCs (Passage 32-40, Harvard University, RRID: CVCL_0057), and FSPS13B hiPSCs (Passage 47-52, a kind gift from L. Vallier). Experiments were carried out on hypothalamic cultures 25–90 days post-differentiation, at which point POMC could be robustly detected by immunocytochemistry. Cultures were maintained in 24-well plates (Corning, Cat# 3524) for proteomic experiments, in 8-well Ibidi dishes (Thistle Scientific, Cat# IB-80826) for confocal imaging, and in Labtek 4-well chamber slides (Sigma–Aldrich, Cat# C6932) for Immunogold EM.

### 2.2 Immunocytochemistry and confocal imaging

Cells were fixed in 4% w/v paraformaldehyde in PBS for 10 min at room temperature. After three washes in TBS, cultures were incubated overnight at 4 °C with primary antibody diluted in 10% normal donkey serum in TBS with 0.1% Triton X-100. Primary antibodies used were raised against MAP2 (1:2,000, Abcam, Cat# ab5392, RRID:AB_2138153), TUJ1 (TUBB3) (1:2,000, BioLegend, Cat# 845502, RRID:AB_2566589), or POMC (A1H5, A3H9, N1C11, used at 1:5,000, developed by Prof. Anne White). Primary antibody was removed by washing three times in TBS. Cells were then incubated in Alexa Fluor-conjugated secondary antibodies (Thermo Fisher Scientific) diluted 1:500 in 10% normal donkey serum in TBS with 0.1% Triton X-100 for 2 h at room temperature. After three washes in TBS, cells were incubated in 360 nM DAPI in TBS, and then washed an additional three times in TBS. Cells were imaged in TBS on a Zeiss LSM 510 confocal microscope. Images were analysed and processed with ImageJ (NIH, RRID:SCR_003070).

### 2.3 Immunogold labelling

hPSC-derived hypothalamic neurons were grown in 4-well Nunc Lab-Tek chamber slides (Sigma–Aldrich, Cat# C6932) as described above. Cells were fixed in 4% paraformaldehyde and 0.5% glutaraldehyde in 0.1 M phosphate buffer (PB) for 1 h at 37 °C. Pre-embedding Immunogold labelling was performed by incubation with either mouse anti POMC A1H5 (1:2,500; developed by Prof. Anne White) or rabbit anti β-endorphin (1:500; Millipore, Cat# Ab5028, RRID:AB_91645) primary antibodies as previously described [41].

### 2.4 Sandwich ELISAs for secreted POMC

ELISAs for secreted POMC were carried out as previously described [37], [38], [42]. Briefly, media from hPSC-derived cortical or hypothalamic neurons (collected after 18 h of incubation with the cells) or supernatant from samples incubated for 10 mins in KCl or ACSF (see Stimulated secretion of POMC peptides section below for details), and human POMC protein standards (concentration range 7.5–600 pM), were diluted in PBS containing 4% BSA, 1% Horse Serum, 50 mg/L Mouse IgG, 500 mg/L Bovine IgG and 0.5% Tween. The diluted samples were then incubated overnight at 4 °C in plates coated with anti-POMC A1A12 antibody (developed by Prof. Anne White). Plates were then rinsed with wash buffer (8.5 mM K_2_HPO_4_, 1.72 mM KH_2_PO_4_, 770 mM NaCl, 0.025% Tween-20, 0.0125% Proclin-300) and then incubated for two hours with agitation in biotinylated N1C11 antibody (developed by Prof. Anne White) at room temperature. After washing, plates were incubated with avidin-HRP for 30 mins at room temperature with agitation. Plates were developed using TMB (Europa Bio-product, Cat# M0701B) for 10–30 mins at room temperature in the dark, and then stopped by addition of 0.5 M HCl. Absorbance at 450 nm was quantified using a plate reader (Tecan M1000 Pro) and then peptide concentration was determined by comparison with the POMC protein standards.

### 2.5 Electron microscopy

Cells were post-fixed in 1% osmium tetroxide and 7% sucrose in 0.1 M PB for 30 min at room temperature, washed in deionized water, and partially dehydrated in 70% ethanol. Cells were then stained in 2% uranyl acetate in 70% ethanol in the dark for 150 min at 4 °C. Cells were further dehydrated in ethanol, and infiltrated overnight in Durcupan ACM epoxy resin (Fluka, Sigma–Aldrich, St. Louis, USA). Following resin hardening, embedded cell cultures were detached from the chamber slide and glued to resin blocks. Serial semi-thin sections (1.5 μm) were cut with an Ultracut UC-6 ultramicrotome (Leica, Heidelberg, Germany) and mounted onto glass microscope slides and lightly stained with 1% toluidine blue. Selected semi-thin sections were glued with Super Glue-3, Loctite (Henkel, Düsseldorf, Germany) to resin blocks and subsequently detached from the glass-slides by repeated freezing (in liquid nitrogen) and thawing. Ultra-thin sections (70–80 nm) were obtained with the ultramicrotome from detached semi-thin sections, and further stained with lead citrate (Reynolds’ solution) [43]. Finally, cells were imaged at 80 kV on a FEI Tecnai G^2^ Spirit transmission electron microscope (FEI Europe, Eindhoven, Netherlands) equipped with a Morada CCD digital camera (Olympus Soft Image Solutions GmbH, Münster, Germany).

### 2.6 Tissue homogenization and peptide extraction from cell pellets and ACSF

To extract and solubilise peptides, cultures were partially dissociated by treatment with 1 mM EDTA in PBS for 10 min. EDTA was then removed, and cells were directly lysed by vigorously pipetting cells with 80% acetonitrile (ACN, Pierce Cat# 51101) as previously described [44], [45]. This lysate was then transferred to a low protein-binding microcentrifuge tube (Sigma, Cat# Z666505), vortexed for 1 min, and pelleted for 15 min at 12,000 g at 4 °C. The supernatant was then transferred to a new low protein-binding microcentrifuge tube or 96-well low protein-binding plate (VWR, Cat# 951032921). In some experiments, cells were lysed in 6M guanidine hydrochloride (GuHCl) [46], [47] (Sigma, Cat# G4505) to aid in peptide extraction [48] and transferred to low-protein-binding microcentrifuge tubes (Sigma, Cat# Z666505). Lysates were snap-frozen on dry ice and thawed on ice three times, before four parts 80% acetonitrile were added and mixed thoroughly. Phases were separated by centrifugation for 5 min at 12,000 g at 4 °C. The aqueous (lower) phase was transferred to a fresh low protein-binding microcentrifuge tube or 96-well plate as described above.

For extraction of peptides from C57BL/6 mouse plasma, 50 μL of plasma was precipitated with 300 μL of 80% ACN in water (v/v) and then centrifuged at 12,000 × *g*. The supernatant was then transferred to fresh low-protein-binding Eppendorf tubes as described above. Both mouse and human samples were either immediately processed, or stored at −80 °C and thawed on ice before being dehydrated in an evaporating centrifuge or with pure nitrogen at 40 °C on an SPE Dry evaporator system (Biotage, Uppsala, Sweden). Dehydrated peptides were reconstituted with 0.1% formic acid in dH_2_O, spiked with stable isotope-labelled internal standards for d-α-MSH(1-13), β-MSH(1-18) and β-EP (1-31), and loaded onto a HLB Prime micro elution plate (Waters, Cat# 186008052) for solid-phase extraction. Plates were washed with 200 μL 0.1% formic acid in dH_2_O (v/v), followed by a wash with 200 μL 1% acetic acid with 5% methanol in dH_2_O (v/v/v). Samples were eluted with 2 × 30 μL of 60% Methanol in dH_2_O with 10% Acetic acid (v/v) and diluted further with 75 μL of 0.1% formic acid in dH_2_O (v/v) and stored at −80 °C until loading onto LC columns for MS/MS.

ACSF from stimulation experiments was collected and immediately snap-frozen on dry ice and stored at −80 °C. Samples were then thawed on ice, followed by spike-in of 500 pg stable-isotope internal standards for α-MSH(1-13), d-α-MSH(1-13), β-MSH(1-18) and direct solid-phase extraction, as described above.

### 2.7 Stimulated secretion of POMC-derived peptides

hPSC-derived neurons were washed three times with HEPES-buffered ACSF consisting of 129 mM NaCl, 5 mM KCl, 1 mM CaCl_2_, 1 mM MgCl_2_, 25 mM HEPES, 11.1 mM Glucose. For experiments in which POMC was detected by ELISA, cells were incubated in ACSF for 10 min at 37 °C. The supernatant was collected and snap-frozen on dry ice. After removing the ACSF, cells were incubated for 10 min at 37 °C in either ACSF or a 129 mM KCl solution consisting of 5 mM NaCl, 129 mM KCl, 1 mM CaCl_2_, 1 mM MgCl_2_, 25 mM HEPES, and 11.1 mM glucose [49], [50] to potently depolarise cells. For experiments in which POMC-derived peptides were detected by LC-MS/MS, we used longer incubation times (90 mins) combined with lower KCl concentrations (30 mM) in order to more gently depolarise cells. Here, cells were incubated in ACSF for 90 min at 37 °C to reduce tonic release, after which the supernatant was collected and snap-frozen on dry ice, and cells were incubated for 90 min at 37 °C in either ACSF or a 30 mM KCl solution consisting of 104 mM NaCl, 30 mM KCl, 1 mM CaCl_2_, 1 mM MgCl_2_, 25 mM HEPES, and 11.1 mM glucose to depolarise cells.

Media supernatants from control ACSF or KCl treatments were stored at −80 °C. To account for slight well-to-well variation in the amount of POMC neurons or POMC-derived peptide production, KCl-induced secretion during the stimulation period (Figure 6) was normalized by subtracting the quantified pre-stimulation value for each POMC-derived peptide from the post-stimulation value.

**Figure 2:**
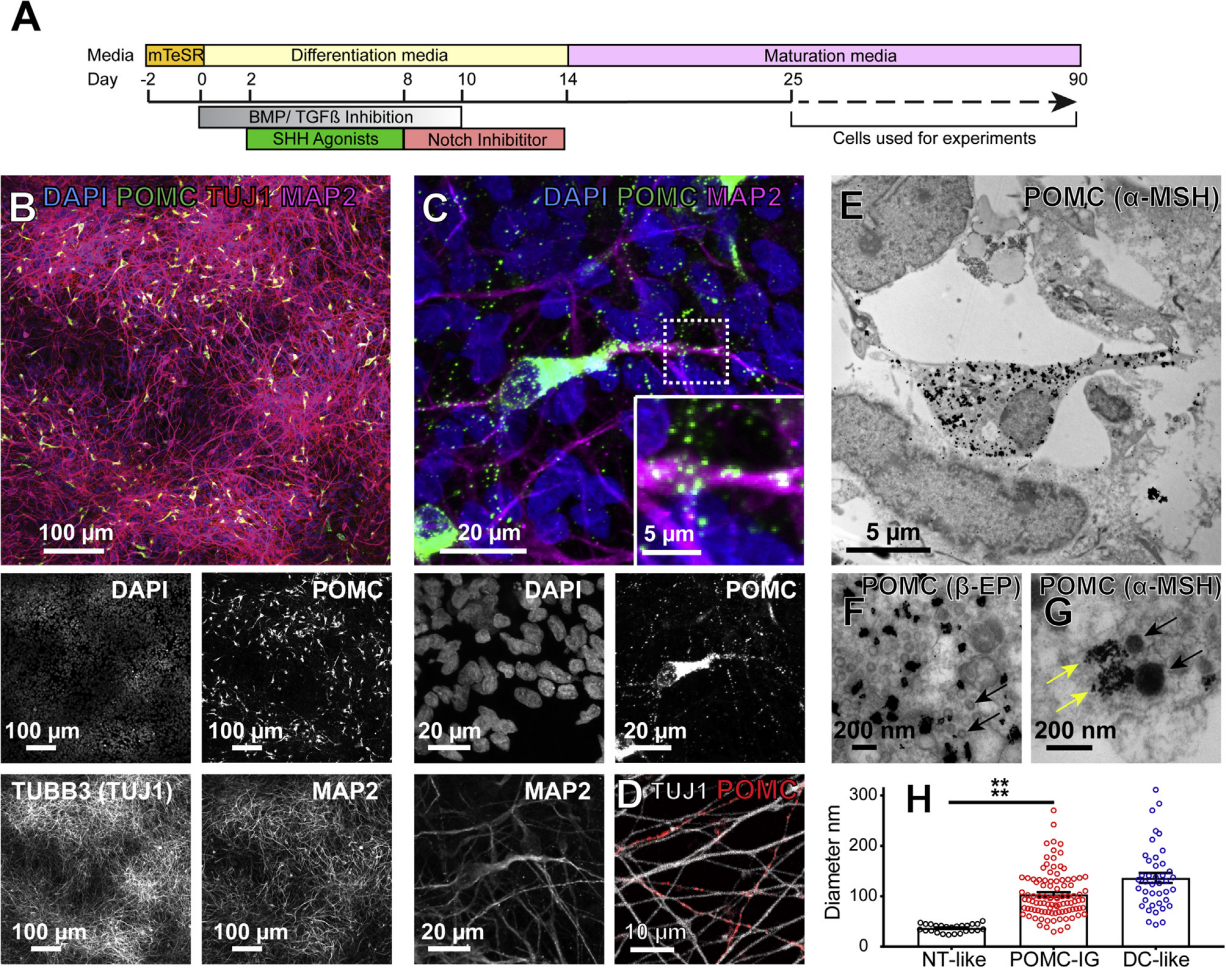
Subcellular localisation of POMC and its derivatives in hPSC-derived hypothalamic neurons. A) Schematic diagram of the differentiation of human pluripotent stem cells into hypothalamic POMC neurons that mature over time in culture. Experiments were carried out between 25 and 90 days post-differentiation. B) Human hypothalamic differentiation yields predominantly neuronal cultures as indicated by immunostaining for Tuj1 and MAP2, of which approximately 6% are immunopositive for POMC. C) Confocal micrographs of punctate POMC-immunoreactive structures localised to cell bodies and neurites (inset). D) Some Tuj1-expressing axon-like processes are strongly immunopositive for POMC (red staining). E-G) Transmission electron micrographs showing specific cytoplasmic and punctate labelling by Immunogold staining for β-EP (F) or α-MSH (G) localised to rounded, vesicle-like structures (G, yellow arrows) in regions with abundant vesicles resembling dense core vesicles (G, black arrows), and adjacent to neurotransmitter-like vesicles (F, black arrows). H) Quantification of the diameters of neurotransmitter-like (NT-like, black circles) clear vesicles, POMC Immunogold-positive (POMC-IG, red circles), and dense core-like (DC-like, blue circles) vesicles in hPSC-derived hypothalamic neurons. Neurotransmitter-like vesicles were significantly smaller than POMC Immunogold-positive structures (p < 0.0001). Error bars show SEM. ****, P < .0001.

**Figure 3:**
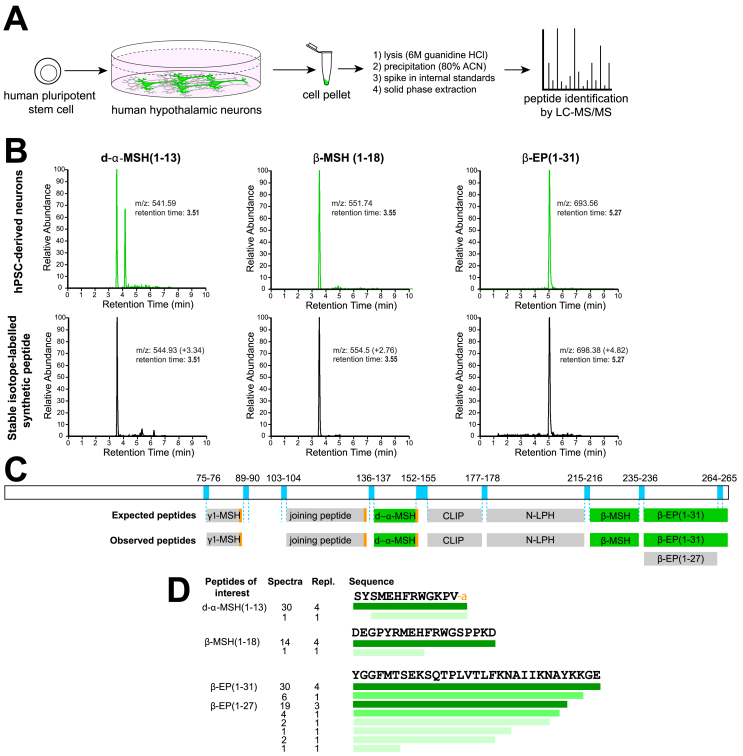
Identification of processed POMC peptides by LC-MS/MS. A) Schematic workflow for the quantification of POMC-derived peptides from hPSC-derived hypothalamic neurons. B) Liquid chromatographs of samples from hPSC-derived hypothalamic neurons (top), compared with stable isotope-labelled synthetic d-α-MSH(1-13), β-MSH(1-18) and β-EP (1-31) (bottom). Note that heavy isotope-labelled reference peptides have identical retention times as endogenous peptides but have slightly higher mass-to-charge (m/z) ratios, and that m/z values measured from Orbitrap and triple quadrupole mass spectrometers may differ. C) Schematic of human POMC protein and relevant dibasic cleavage sites (blue), expected POMC-derived peptides, and those peptides detected in hPSC-derived hypothalamic cultures by LC-MS/MS. Detected peptides included γ_1_-MSH (Lys- γ_1_-MSH), d-α-MSH(1-13), β-MSH(1-18) and β-EP (1-31). Quantified peptides are indicated in green. D) Summary of POMC-derived peptides detected by LC-MS/MS, where the colour intensity represents the relative abundance of each peptide species. Repl., replicates. Other abbreviations are as in Figure 1.

**Figure 4:**
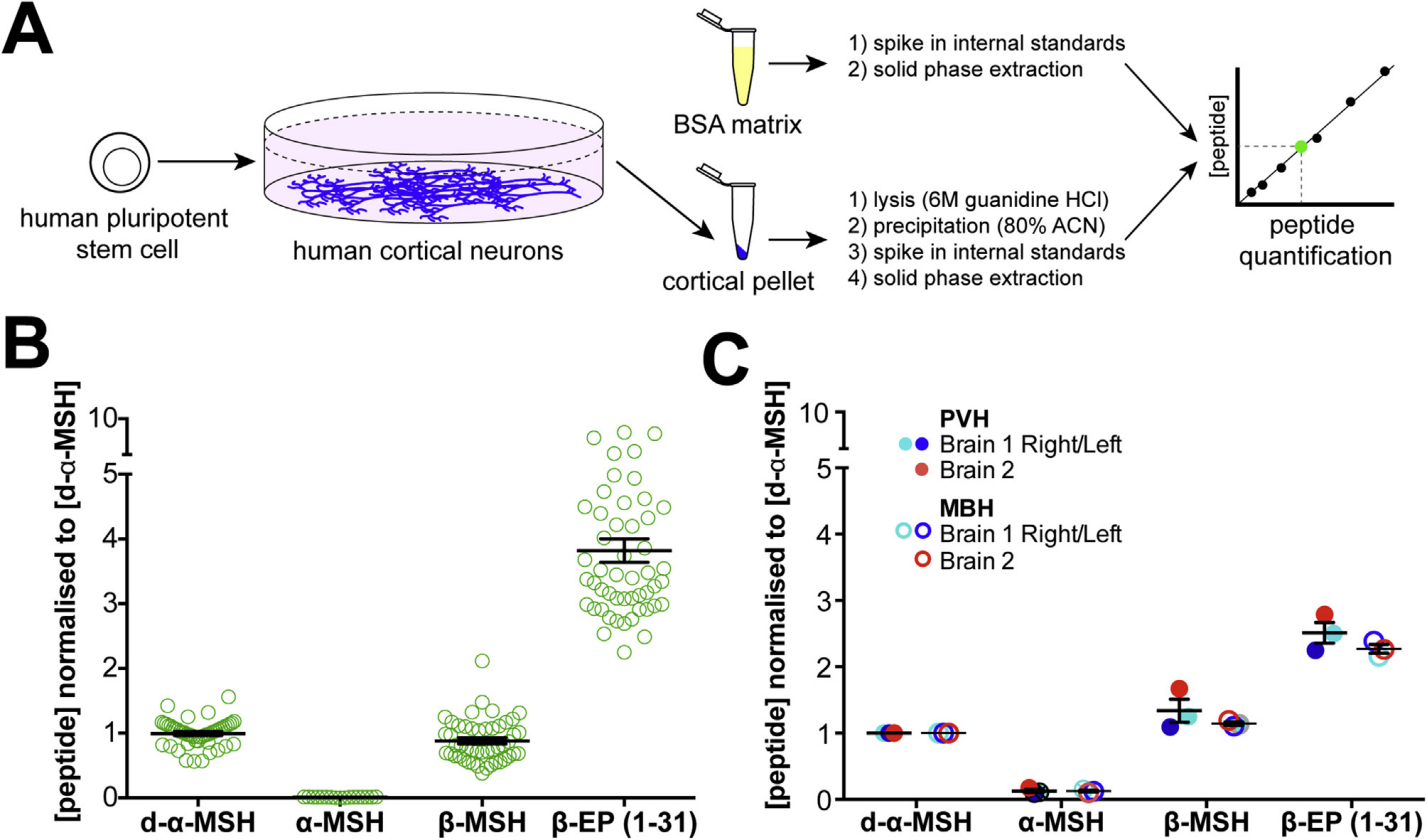
Quantification of POMC-derived peptides in hPSC-derived neurons and primary human hypothalamus. A) Schematic diagram of standard curve generation for peptide quantification. B) LC-MS/MS-based quantification of d-α-MSH, α-MSH, β-MSH and β-EP peptides in hPSC-derived POMC neurons. N = 5 independent experiments with 3–8 replicates per experiment. Concentrations were converted to molarity and then normalised to the mean concentration of d-α-MSH for each technical replicate. Error bars show SEM. C) Quantification of POMC-derived peptides in primary human brain samples. Note that while α-MSH was clearly detected in PVH Brain 1 Right and Left and MBH Brain 1 Right, the concentration was below the assay's limit of accurate quantification. N = 3 independent samples from N = 2 brains per brain region. PVH, dissected region encompassing the paraventricular hypothalamus; MBH, dissected region encompassing the mediobasal hypothalamus. Error bars show SEM.

**Figure 5:**
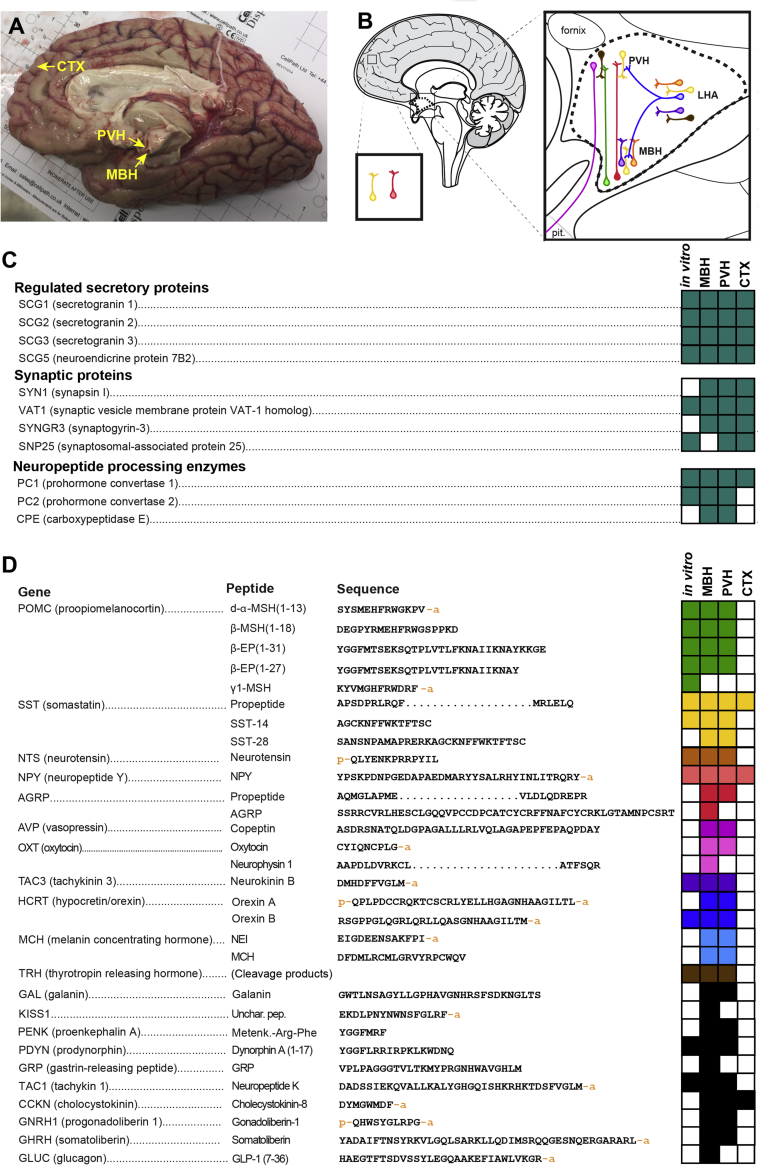
Characterisation of the peptidome of the human hypothalamus. A) Sagittal view of the right hemisphere of a post-mortem human forebrain used in this study. Regions of approximately 3 × 3 × 3 mm from the indicated brain regions were dissected for analysis. B) Colour-coded schematic diagram of several neuropeptidergic cell types of interest. C) Summary of peptides associated with the regulated secretory pathway, synaptic proteins, and neuropeptide processing enzymes detected in hPSC-derived hypothalamic neurons *in vitro* or in the MBH, PVH, or CTX of the human brain. Filled boxes represent peptides corresponding to the indicated gene that were detected and automatically identified. D) Summary of select neuropeptides detected in hPSC-derived hypothalamic neurons or in the human brain. Sequences too long to readily display are denoted by an ellipsis. CTX, cortex (orbitofrontal gyrus); LHA, lateral hypothalamic area; MBH, mediobasal hypothalamus; Pit., pituitary gland; PVH, paraventricular nucleus of the hypothalamus, Unchar. Pep., uncharacterised peptide. An adjoining ‘p’ on the amino acid sequences denotes a pyroglutamate residue, while an ‘a’ denotes an amide group. N = 4 independent samples from N = 3 unrelated fresh post-mortem brains.

**Figure 6:**
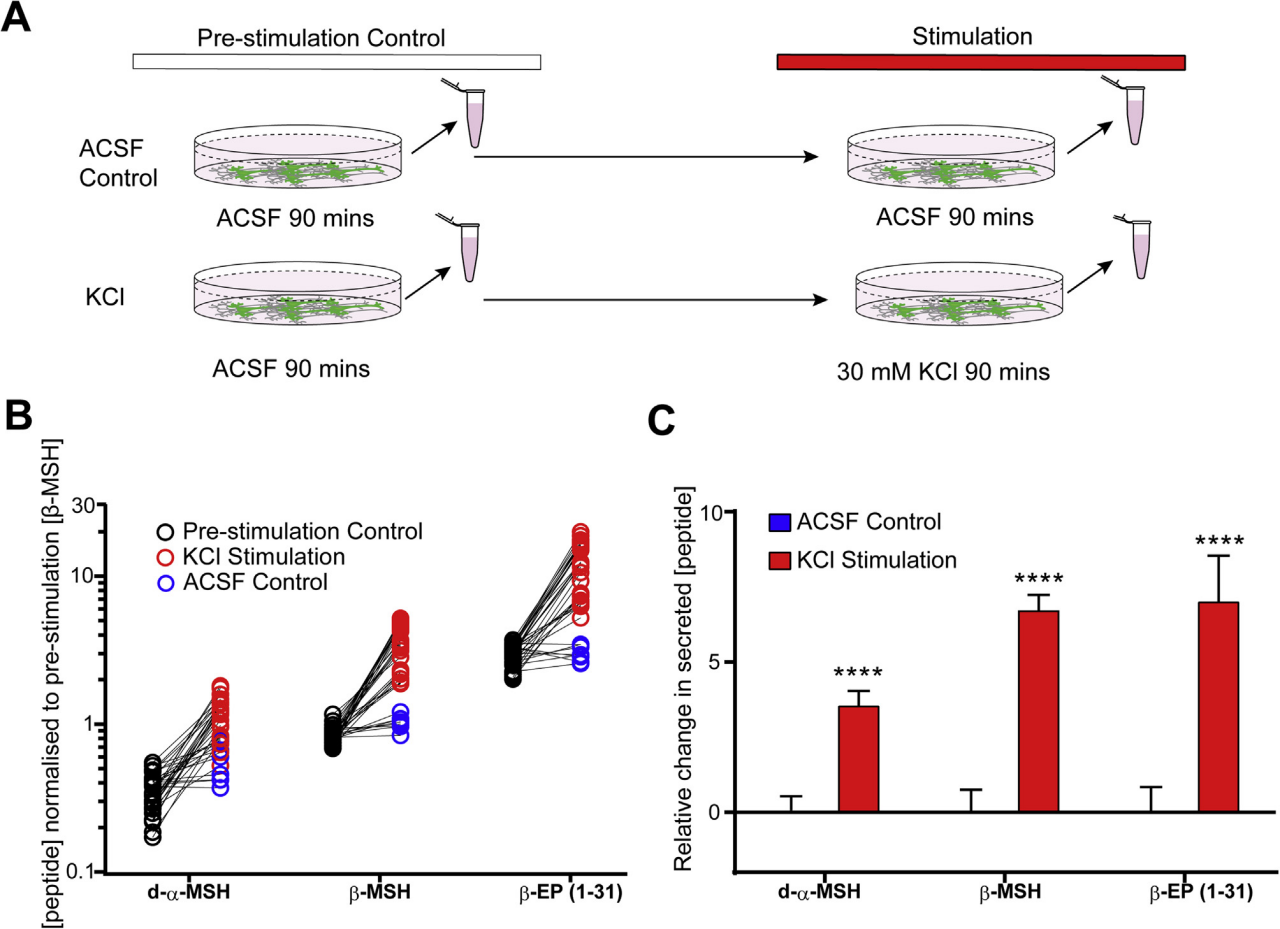
Regulated secretion of POMC-derived peptides. A) Experimental schematic for quantifying the stimulated secretion of POMC-derived peptides in hPSC-derived hypothalamic neurons by LC-MS/MS. B) Secreted d-α-MSH, β-MSH and β-EP (1-31) peptides in pre-stimulation controls (black circles), 30 mM KCl stimulations (red circles) and ACSF controls (blue circles). Molar peptide concentrations were normalised to the mean pre-stimulation β-MSH concentration. N = 4 independent experiments with 4–8 technical replicates per experiment. C) KCl-induced changes in secreted peptide concentration were calculated from data shown in (B) for each technical replicate by subtracting the pre-stimulation peptide concentrations from the ACSF control or KCl stimulation condition, and normalising to mean concentrations seen in ACSF control-stimulated cultures. KCl stimulation significantly (P < 3 × 10^−5^) increased the concentrations of d-α-MSH, β-MSH and β-EP (1-31). KCl, potassium chloride. Error bars show SEM. ****, P < .0001.

### 2.8 Regulation of MSH concentration by exogenous factors

hPSC-derived hypothalamic neuronal cultures were dissociated after D25, at which point POMC neurons could clearly be visualised by immunoctyochemistry, and re-plated in parallel cultures as previously described [32]. After 24 h of recovery, media was changed and cultures were treated for either 24 h (n = 1 cell line, 2 experiments) or 9 days (n = 2 cell lines, 1 experiment) with experimental agents. These included leptin (PeproTech, Cat# 300-27) reconstituted in PBS + 0.1% BSA to a final concentration of 10 ng/mL, 100 ng/mL, 1 μg/mL or vehicle (PBS + 0.1% BSA), and Furin Inhibitor 1 (Cayman Chemical Co., Cat# B00165) reconstituted in DMSO and added to a final concentration of 25 μM or vehicle (DMSO to 0.1% final concentration). Cultures were then processed for LC-MS/MS as described above.

### 2.9 Peptide discovery by LC-MS/MS

Peptide extracts were run using nano-flow-based separation and electrospray approaches on a Thermo Fisher Ultimate 3000 nano-LC system coupled to a Q Exactive Plus Orbitrap mass spectrometer (ThermoScientific, San Jose, USA) as described previously [44]. Extracts were injected at a flow rate of 30 μL/minute onto a peptide trap column (0.3 × 5 mm; ThermoFisher Scientific), washed for 15 min, and switched in line with a nano-easy column (0.075 × 250 mm; ThermoFisher Scientific) flowing at 300 nL/minute. Both nano and trap column temperatures were set at 45 °C during the analysis. The buffers used for nano-LC separations were A: 0.1% formic acid in water (v/v) and B: 0.1% formic acid (v/v) in 80:20 ACN/water. Initial starting conditions were 2.5% B (equating to 2% ACN), and held for 5 min. A ramp to 50% B was performed over 135 min, followed by a wash with 90% B for 20 min before returning to starting conditions for 20 min, totalling an entire run time of 190 min. Electrospray analysis was performed using a spray voltage of 1.8 kV, the tune settings for the MS used an S-lens setting of 70 v to target peptides of higher m/z values. A full scan range of 400–1600 m/z was used at a resolution of 75,000 before the top 10 ions of each spectrum were selected for MS/MS analysis. Existing ions selected for fragmentation were added to an exclusion list for 30 s.

### 2.10 Peptide identification using peaks

The acquired LC-MS/MS files were searched using Peaks 8.5 software (Waterloo, ON, Canada) against the *Homo sapiens* Swissprot database for human samples, or the *Mus musculus* Swissprot database for mouse plasma samples (06-May-2016, 26-October-2017). A no-digest setting was used, which enabled peptides of up to 65 amino acids in length to be matched, and precursor and product ion tolerances were set at 10 ppm and 0.05 Da, respectively. A fixed post-translational modification of carbamidomethylation was applied to cysteine residues, whilst variable modifications included methionine oxidation, N-terminal pyro-glutamate, N-terminal acetylation and C-terminal amidation. A minimum of 1 unique peptide and a false discovery rate value of 1% was used to filter the results.

### 2.11 Peptide quantification by LC-MS/MS

Calibration standards of synthetically-produced α-MSH(1-13), d-α-MSH(1-13), β-MSH(1-18) and β-EP (1-31) (Bachem, Bebendorf, Switzerland) were diluted to 10-10,000 pg/mL or 100-100,000 pg/mL in a matrix of 0.001% BSA in 0.1% formic acid (v/v), or in lysates from cultured cortical neurons that contained no detectable POMC peptides to mimic the protein and lipid composition from hypothalamic neuron extracts. Calibration standards were then spiked with 500 pg custom-ordered stable-isotope internal standards (Cambridge Research Biochemical Ltd.) for:

d-α-MSH(1-13): SYSMEH-[U-^13^C_9_,^15^N-Phe]-RWGKPV-amide

β-MSH(1-18): DEGPYRMEH-[U-13C9,15N-Phe]-RWGSPPKD-acid

β-EP (1-31): YGGFMTSEKSQTP-[U-^13^C_6_,^15^N-Leu]-VT-[U-^13^C_5_,^15^N-Leu]-[U-^1 3^C_9_,^15^N-Phe]-FKNAIIKNAYKKGE-acid

Samples were extracted using the solid phase extraction method described above. LC-MS/MS instrumentation used for the quantitation of the POMC-derived peptides included a H-Class Acquity (Waters) attached to a TQ-XS triple quadrupole mass spectrometer (Waters). Sample (10 μL) was injected onto a 2.1 × 50 mm 1.8 μm particle HSS T3 Aquity column held at 60 °C and flowing at 700 μL/minute. Gradient starting condition were 87.5% A (0.1% formic acid in water v/v) and 12.5% B (0.1% formic acid in ACN). Starting conditions were held for 30 s before raising to 30% B over 2.5 min. The column was flushed with 90% B for 1.1 min before returning to starting conditions. The total time of each analysis was 5 min, with the first 1.1 min and last 1.8 min diverted to waste. Mass spectrometry conditions involved targeting four POMC-derived peptides, and the transition and collision energy details for each peptide, along with associated internal standards are given in Table 1.

**Table 1:** Peptide transitions (Q1, Q3), collision energies and dwell time for POMC peptide standards and internal standards. m, mass; z, charge; eV, electron volts; ms, milliseconds; IS, internal standard.

Peptide	Q1 (*m/z*)	Q3 (*m/z*)	Collision energy (eV)	Dwell time (ms)
d-α-MSH	406.55	458.13	12	40
d-α-MSH IS	409.05	461.56	12	40
α-MSH	555.98	686.75	19	40
β-MSH	552.1	654.08	20	40
β-MSH IS	554.6	567.41	20	40
β-Endorphin (1-31)	693.62	825.89	22	40
β-Endorphin (1-31) IS	698.52	832.19	22	40

The source parameters used included a positive electrospray ion spray voltage of 3.0 kV, gas flow of 1000 L/hour, desolvation temperature of 600 °C and a cone voltage of 40 V. Peptide peak areas were integrated using the TargetLynx program associated with Masslynx V 4.2 (Waters), and peptide peak areas were ratiometrically compared against their corresponding stable isotope-labelled internal standard peptide. The POMC-derived peptide peak area ratio concentrations in extracted samples were compared against the calibration lines to assign concentration values. The peptide calibration lines showed consistent results over a thousand-fold range of peptide concentrations (R^2^ > 0.98 for all peptides).

### 2.12 Human brain samples

Fresh human brain samples were obtained from three donors of unknown body mass index who had previously given informed consent via the Cambridge Brain Bank after ethical committee approval. Samples were from a 75-year-old woman, 81-year-old woman with no medical history or obvious pathological evidence of neurodegeneration, and from a 79-year-old man with signs of multiple system atrophy. After death, brains were dissected from the skull and stored in phosphate buffered saline (PBS) at 4 °C for 24 h, 57 h, and 48 h respectively, until the time of dissection. After dissection, samples were immediately snap frozen in 6 M guanidine hydrochloride, stored at −80 °C, and then processed and analysed by LC-MS/MS. Dissected brain regions of approximately 3 × 3 × 3 mm encompassed the mediobasal hypothalamus (MBH) that should contain POMC cell bodies, the paraventricular nucleus of the hypothalamus (PVH) that should contain POMC neuron axon terminals, and a small piece of the orbitofrontal gyrus of the cerebral cortex (CTX) extending through all cortical layers. Immediately after dissection, primary samples were disrupted in 6M GuHCl at 4 °C in Lysing Matrix D tubes (MPbio, Cat# 116913100) to stabilise and extract peptides, and then fully homogenized using a Fastprep 24 5G homogeniser (MPbio) using four 40 s runs at 6 m/s separated by 5 min of cooling on ice between each homogenisation cycle. Samples were stored at −80 °C or processed and analysed immediately as described above.

### 2.13 Statistical analysis

**Figure 7:**
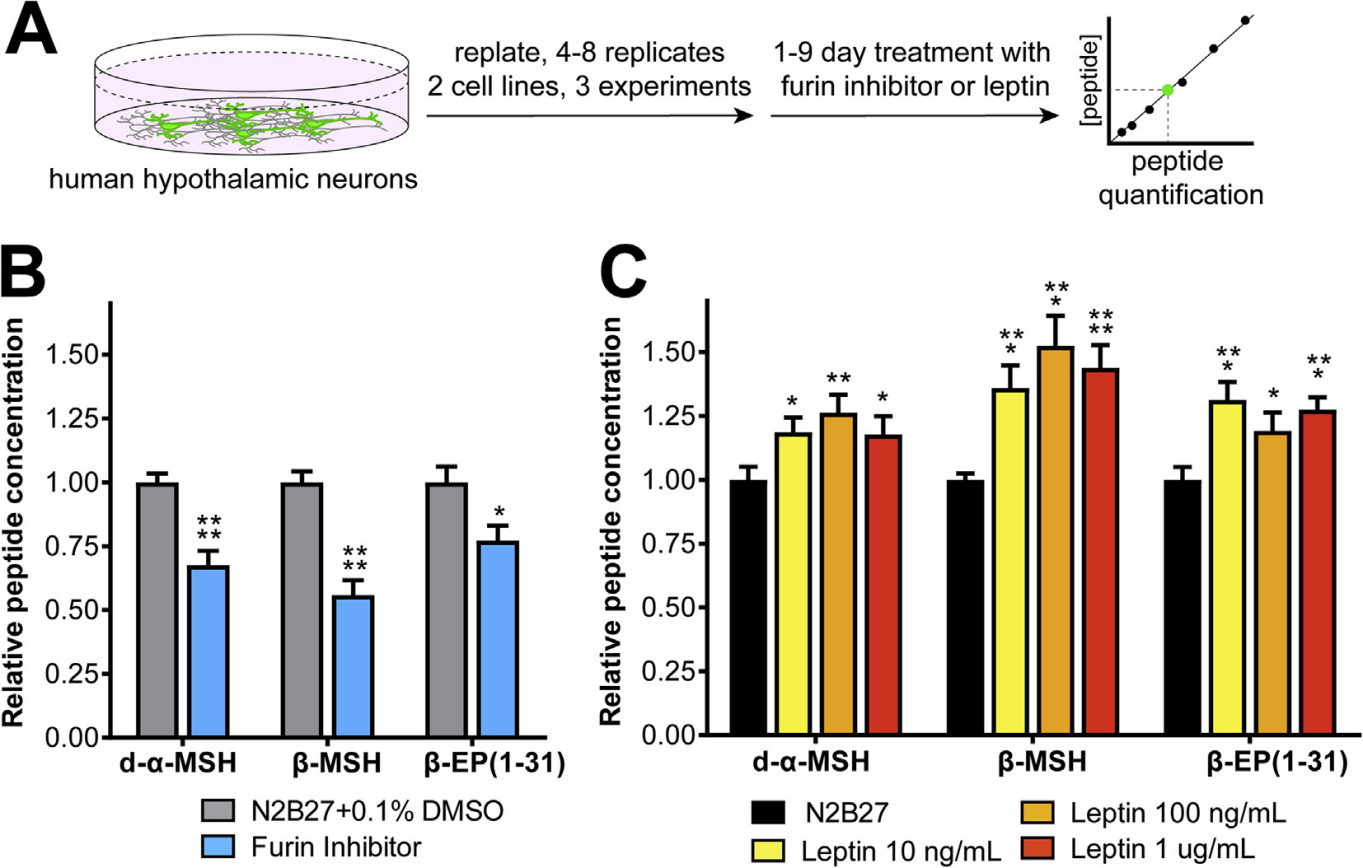
Regulation of POMC processing *in vitro*. A) Experimental schematic for measuring the regulation POMC and its derivates by leptin or Furin inhibitor I, which blocks prohormone convertases. B) Treatment with 25 uM Furin Inhibitor 1 (blue) significantly reduced d-α−MSH and β-MSH (P < 0.0001), and β-EP (P < 0.05) concentrations. C) Treatment with recombinant human leptin significantly increased the measured concentrations of d-α−MSH, β-MSH, and β-EP at all concentrations tested. N = 3 independent experiments with 4–8 technical replicates per experiment. Error bars show SEM. *, P < 0.05; **, P < 0.01; ***, P < 0.001; ****, P < 0.0001.

Statistical analyses in Figures 2H, 6C, 7B,C, S1E were carried out using Prism (GraphPad, RRID:SCR_002798). Significance was calculated using the Holm-Sidak method, with alpha 0.05. Error bars in Figures 2H, 4B,C, 6C, 7B,C, S1C,E, S3B show the standard error of the mean (SEM).

### 2.14 LC-MS/MS data

The mass spectrometry peptidomics data have been deposited to the ProteomeXchange Consortium via the PRIDE partner repository with the dataset identifier PXD008795 and 10.6019/PXD008795 (http://proteomecentral.proteomexchange.org). Explanation of file names is summarised in Table 2.

**Table 2:** Summary of post-mortem human brain samples files names and samples types for the peptidomics data deposited on the PRIDE partner repository.

Sample ID	Sample Type	Data file name	Age	Sex	Cause of Death	Post-Mortem Interval	LC-MS Analysis type
Brain 1 Left, Right	MBH, PVN, CTX	n/a	79	Male	Multiple system atrophy	48 h	Quantitative and Nano-flow
Brain 2 Right	MBH, PVN, CTX	n/a	81	Female	Leukaemia	60 h	Quantitative and Nano-flow
Brain 3 Left	MBH	ARC brain 1	75	Female	Kidney Cancer	24 h	Nano-flow
Brain 3 Right	MBH	ARC brain 2	75	Female	Kidney Cancer	24 h	Nano-flow
Brain 3 Left	PVN	PVN brain 1	75	Female	Kidney Cancer	24 h	Nano-flow
Brain 3 Right	PVN	PVN brain 2	75	Female	Kidney Cancer	24 h	Nano-flow
Brain 3 Cortex	Cortex, orbitofrontal gyrus	CTX Brain 1	75	Female	Kidney Cancer	24 h	Nano-flow
hPSC-derived cortical neurons	Cultured cortical neurons (WA09 ES line)	Cortex neurons ACN	n/a	n/a	n/a	n/a	n/a
hPSC-derived hypothalamic neurons	Cultured hypothalamic neurons (HUES9 ES line)	POMC neuron GuHCL	n/a	n/a	n/a	n/a	n/a

## 3 Results

### 3.1 Human hypothalamic neurons appropriately traffic and secrete POMC *in vitro*

To determine if *in vitro*-derived human POMC neurons could be a suitable model system for studying POMC processing, we differentiated human embryonic stem cells (hESCs) and human induced pluripotent stem cells (hiPSCs) into hypothalamic neurons as previously described [31], [32] (Figure 2A). After 28 days of differentiation, cultures expressed hypothalamic marker genes such as NKX2.1, and predominantly contained cells with neuronal morphology that expressed the neuronally-enriched proteins tubulin beta 3 class III (TUBB3, Tuj1) and microtubule associated protein 2 (MAP2) (Figure 2B). We observed that 6.5 ± 0.76 percent of neurons were strongly immunoreactive for POMC, as visualised by several distinct antibodies (Figure 2B–D).

Since POMC is packaged into dense core vesicles as it is progressively processed [51], [52], [53], [54], we examined the subcellular localization of POMC-immunoreactive epitopes by high-magnification immunofluorescent confocal microscopy and found that they localised to punctate structures within the cell body and in MAP2 and Tuj1-expressing neurites (Figure 2C,D). To ask if these structures corresponded to dense-core vesicles, we performed Immunogold labelling with antibodies recognising epitopes corresponding to sequences in α-MSH or β−EP and their precursor peptides, and examined samples by transmission electron microscopy. Immunogold particles (Figure 2E–G) localised to rounded structures 103.5 ± 4.5 nm in diameter that were similar in appearance and localisation (Figure 2G, yellow arrows) to electron-opaque dense-core vesicles (Figure 2G, black arrows) that averaged 136.4 ± 9.84 nm in diameter (Figure 2H). In contrast, numerous clear neurotransmitter-like vesicles (Figure 2F, arrows) were significantly (P < 0.0001) smaller in diameter at 36.6 ± 1.6 nm (Figure 2H). Overall, these measurements are similar to those reported *in vivo* for neurotransmitter-containing vesicles (30–50 nm) and dense-core vesicles (100–250 nm) in both rodents [55], [56], [57], [58] and humans [59], [60]). These results suggest that POMC and/or its derivative peptides are likely packaged into vesicles that mature into dense-core vesicles.

Since these findings suggested that POMC might be secreted, we analysed the culture media collected from hPSC-derived cortical or hypothalamic cells using a sensitive (detection limit ∼7.5 pM) sandwich ELISA. This assay detects epitopes in ACTH(10-18) and NPP [37], [38], [42], both of which are present in full-length POMC and in pro-ACTH (Figures 1, S1A,B). We could readily detect these species in media from hypothalamic cultures but not from cortical cultures (Fig. S1C), consistent with their constitutive secretion and/or secretion in response to spontaneous neuronal activity present during standard culture conditions. To test if secretion might be stimulated by depolarisation, we treated cultures with potassium chloride (KCl) (Fig.S1D) and observed a significant (P < 0.01) 6.3 ± 0.16-fold increase in secreted POMC and/or pro-ACTH (Fig. S1E).

### 3.2 hPSC-derived hypothalamic neurons produce β-MSH(1-18) and other POMC-derived peptides

To identify which POMC-derived peptides were produced in human hypothalamic neurons, we extracted peptides using either 80% acetonitrile (ACN) or 6M guanidine hydrochloride (GuHCl) and ACN, followed by solid-phase extraction and analysis by nanoflow LC-MS/MS [61] (Figure 3A). We observed similar types of peptides using these two extraction methods, but found that GuHCl treatment increased both the number of unique peptides and the depth of spectra observed per peptide (Table S1).

To test whether hPSC-derived hypothalamic neurons processed POMC into peptides important for energy homeostasis, we obtained custom-synthesised d-α-MSH(1-13), β-MSH(1-18) and β-EP (1-31) with specific amino acids substituted for ^13^C and ^15^N stable isotopically labelled equivalents, and spiked these internal standards into cell lysates during peptide extraction (Figure 3A). A single d-α-MSH standard was used for quantification of both d-α-MSH and α-MSH to ensure accurate relative quantification, since these peptides have very similar chemical properties. Stable isotope-labelled standards have shifted mass-to-charge ratios (m/z) compared with endogenous peptides, but are otherwise chemically identical, resulting in identical column retention times during liquid chromatography (Figure 3B).

We then identified peptides present in samples from two unrelated hESC lines and one hiPSC line using an automated peptide identification pipeline (see Materials and Methods). We consistently observed POMC-derived peptides in hPSC-derived hypothalamic cultures, constituting 3.35 ± 1.87% of all peptide alignments (Table S2), but never observed them in hPSC-derived cortical-like cells. The vast-majority of unique spectra for POMC-derived peptides of interest (50/69, 72.4%) were flanked by dibasic residues that are targeted by POMC-processing enzymes (Figure 3C), and some peptides were C-terminally amidated as is seen *in vivo* (e.g. d-α-MSH), indicating that the observed peptides were unlikely to be random degradation products (Figure 3D). We identified the POMC-derived neuropeptides d-α-MSH, β-MSH and β-EP (1-31) as well as β-EP (1-27), γ_1_-MSH (Lys-γ_1_-MSH) [62], CLIP, N-LPH, and joining peptide (Figures 1 and 3C). These results extend a previous report on hPSC-derived peptide production [63] by providing the specific sequences and post-translational modification patterns of these peptides and establishing that β-MSH(1-18) is produced by human hypothalamic neurons.

We were surprised to readily detect spectra for β-MSH(1-18), the existence of which has been disputed [21], whereas we did not consistently detect spectra for α-MSH(1-13), which has been the subject of intense study [7], [8], [30], [64], [65] and is thought to be more stable than d-α-MSH [30]. Upon manual review of the raw data, we could indeed find peaks with m/z and column retention times appropriate for α-MSH. We therefore wondered what the relative concentrations of POMC-derived peptides relevant for human energy homeostasis might be *in vitro* and *in vivo*.

### 3.3 d-α-MSH(1-13), β-MSH(1-18), and β-EP(1-31) are produced in excess of α-MSH(1-13)

To address this question, we developed quantitative assays [44] for α-MSH(1-13), d-α-MSH(1-13), β-MSH(1-18) and β-EP (1-31) (Figure 4A) by generating calibration lines of these synthetic peptides that enabled accurate (R^2^ > 0.98) peptide quantification over a broad range and down to at least 10 pg/ml (2.9–6 pM) (Fig. S2). We then compared extracted samples from hPSC-derived hypothalamic neurons to the calibration line, using the stable isotopically labelled internal standards as a normalising factor for each peptide.

In hPSC-derived hypothalamic cultures, we found that target peptides were typically present at concentrations greater than 540 pM, well above the quantification limit of the assay. Upon comparing the relative concentrations of these peptides across four independent experiments, we found that the concentrations of d-α-MSH and β-MSH were approximately equimolar (1.00 ± 0.03: 0.88 ± 0.05) (Figure 4B). In contrast, we observed α-MSH concentrations to be approximately one hundredth the concentration of d-α-MSH (0.009 ± 0.001) and often near the assay's lower limit for accurate quantification, whereas β-EP (1-31) concentrations were 3.82 ± 0.18- fold higher than that of d-α-MSH (Figure 4B).

The analysis of hPSC-derived hypothalamic cultures yielded many results consistent with what has been described in other experimental systems, but we were surprised by the dramatically higher abundance of both d-α-MSH and β-MSH relative to α-MSH, and the even higher concentrations of β-EP. To test whether observations made *in vitro* were predictive of the concentrations of these peptides *in vivo*, we therefore obtained three fresh post-mortem human brain samples and dissected regions encompassing the mediobasal hypothalamus (MBH), the paraventricular nucleus of the hypothalamus (PVH), and a small piece of the orbitofrontal gyrus of the cerebral cortex (CTX) from each brain (Figure 5A,B). By nanoflow LC-MS/MS analysis of these brain samples, we identified an average of 2089 ± 854 peptides from 4472 ± 2355 spectra per sample, corresponding to 382 ± 86 unique proteins, comparable to what we observed in hPSC-derived hypothalamic neuronal cultures (2164 ± 691 peptides, 4827 ± 1642 spectra, 588 ± 170 unique proteins per sample) (Table S2). In each sample, we observed peptide fragments of proteins associated with synaptic function and regulated secretion, indicating efficient peptide extraction. In primary human hypothalamic samples, we detected fragments of several key enzymes involved in neuropeptide processing, including PCSK1 (PC1/3), PCSK2 (PC2) and CPE (Figure 5C). Primary human MBH and PVH samples contained the POMC-derived peptides α-MSH(1-13), d-α-MSH(1-13), β-MSH(1-18) and β-EP (1-31), along with a broad array of other neuropeptides such as agouti-related peptide (AGRP) (Figure 5D). Many of the peptides identified in the human brain were also present with identical structures and post-translational modifications in hPSC-derived hypothalamic neurons (Figure 5D).

Next, we added stable-isotope-labelled internal standards, applied the quantitative LC-MS/MS quantification methods described above, and found that β-MSH was indeed present in the adult human MBH and PVH at concentrations comparable to that of d-α-MSH (Figure 4C). We also found that β-EP (1-31) was present at approximately 2- to 3-fold higher concentrations than d-α-MSH or β-MSH, while concentrations of acetylated α-MSH concentrations were at least 6-fold lower than those of either d-α-MSH or β-MSH, and were often below the limit for accurate quantification (Figure 4C).

To test whether human α-MSH might be preferentially lost during solid phase extraction, we spiked POMC peptide standards into cellular extracts from hPSC-derived cortical neurons (Fig. S3A) and found that concentrations of POMC-derived peptides were similar before and after solid-phase extraction (median ratio 1:0.99), indicating minimal sample loss or peptide-specific extraction biases (Fig. S3B). In addition, we found that acetylated POMC-derived peptides, including α-MSH, were readily detectable from mouse plasma using the same peptide extraction and LC-MS/MS methods used to analyse human neuronal samples, ruling out any potential detection bias (Fig. S4) [66]. To test whether the higher concentration of β-EP (1-31) relative to d-α-MSH(1-13) and β-MSH(1-18) could be explained by technical artefacts, such as degradation of the standard peptide, we acquired a fresh stock of synthetic β-EP (1-31) and found good agreement (within 16%) with our previous measurements, indicating that the substantially higher concentrations of β-EP (1-31) we observed were not likely due to degradation of the stock peptide.

Overall, the broad agreement between findings from our *in vitro* culture system and primary human brain tissue demonstrate that hPSC-derived hypothalamic neurons have predictive value and suggest that they might also provide insights into the regulation of POMC expression, processing, and secretion.

### 3.4 Human hypothalamic neurons secrete POMC-derived peptides upon depolarisation

To test whether hPSC-derived hypothalamic cultures secrete POMC-derived melanocortins, we replaced the culture media with artificial cerebrospinal fluid (ACSF) and analysed the ACSF by quantitative LC-MS/MS after a brief incubation period. We readily observed d-α-MSH, β-MSH and β-EP (but not acetylated α-MSH) at concentrations within the linear range for LC-MS/MS-based quantification indicating that POMC-derived peptides are robustly secreted (Figures 6A,B, S2).

Since POMC-derived peptides were localised to dense core vesicle-like structures in hPSC-derived hypothalamic neurons (Figure 2F–H), we hypothesised that their secretion might be enhanced by depolarisation. Following ACSF pre-stimulation, we therefore switched cultures to either ACSF (control) or to ACSF containing 30 mM KCl to induce depolarisation (Figure 6A) and found that KCl treatment led to significantly (P < 0.0001) higher concentrations (3.5- to 8.5-fold) of POMC-derived peptides (Figure 6C), suggesting robust stimulated secretion. The molar ratios of secreted d-α-MSH: β-MSH: β-EP in both ACSF and KCl-treated samples were approximately 0.4: 1.0: 3.7 (Figure 6B), broadly resembling the ratios observed in cell lysates (approximately 1.1: 1.0: 4.3) (Figure 4B) with the exception of d-α-MSH, which appeared to be less abundant in the supernatant than in cell lysates. Together, these results suggest that POMC-derived peptides were appropriately trafficked into regulated secretory vesicles (Figure 2C–H) and secreted upon depolarisation.

### 3.5 Leptin increases MSH and β-EP concentrations in human hypothalamic neurons

The regulation of POMC production or processing can alter the pool of neuropeptides available for secretion, which could in turn modulate the downstream effects of POMC-derived peptides on energy homeostasis. Since leptin stimulates POMC neurons [67] and induces POMC gene expression in mice [28], we hypothesized that treatment with leptin might alter the concentration of POMC-derived peptides in human hypothalamic neurons.

As the concentrations of α-MSH were close to the limit of accurate quantification, we selected the more abundant peptides d-α-MSH, β-MSH and β-EP to test this hypothesis. First, to investigate whether changes in neuropeptide processing were detectable by our methods, we added either vehicle or a broad-spectrum inhibitor of prohormone convertases (25 μM Furin Inhibitor 1) to hPSC-derived hypothalamic neurons (Figure 7A). We then extracted peptides from cell lysates, added internal standards, performed quantitative LC-MS/MS, and observed a 23–45% decrease in the measured concentration of d-α-MSH (P < 0.0001), β-MSH (P < 0.0001) and β-EP (P < 0.05) in drug-treated cultures relative to vehicle controls (Figure 7B). Next, we treated cultures with either vehicle or 10 ng/mL (625 pM), 100 ng/mL (6.25 nM) or 1 μg/mL (62.5 nM) human leptin and observed a significant (P < 0.05 to P < 0.0001) 18–52% increase in the concentration of all tested POMC-derived peptides at all tested leptin concentrations (Figure 7C). These results indicate that dynamic changes in POMC peptide concentration are detectable *in vitro* by quantitative proteomic methods, and that cultured human hypothalamic neurons functionally respond to physiologically-relevant concentrations of leptin by altering POMC production and/or processing.

## 4 Discussion

In this study, we used LC-MS/MS to identify and quantify peptides in hPSC-derived neurons and in the human brain. To quantify POMC-derived peptides of interest, we developed quantitative LC-MS/MS-based methods with the aid of stable isotope-labelled internal standards. These methods had comparable sensitivity to most ELISAs and greater specificity, since antibodies may recognise multiple similar forms of a peptide [68], [69], [70]. Our analysis revealed several noteworthy findings.

First, we found that key POMC-derived peptides were appropriately processed *in vitro* in all hPSC lines analysed and had identical structures to those identified *in vivo*, including post-translational modifications. Although we typically observed over 2000 unique peptides per sample, these results are not exhaustive, since degradation in the post-mortem interval may have affected the peptide concentrations measured in primary human brain samples, larger peptides may be precipitated by ACN [71], and glycosylated peptides are not readily detected by the automated analysis pipeline we employed (see Materials and Methods).

We demonstrate using quantitative techniques with structural-level resolution that β-MSH is present at substantial concentrations in both hPSC-derived neurons and in the human hypothalamus. These data are consistent with previous reports that used distinct methodologies. In particular, immunohistochemistry for β-MSH is suggestive but likely fails to discriminate between epitopes unique to β-MSH and those present in its precursors and therefore is open to multiple interpretations [18]. Similarly, HPLC has been used to demonstrate the presence of peptides with chemical properties consistent with β-MSH [15], but this technique does not provide sequence information and it has been suggested that the observed 18- and 22-amino-acid species of β-MSH might be artefacts generated during peptide isolation [20], [21], [22], [72]. In our LC-MS/MS studies, we were able to directly observe peptide sequences, including degradation products, and found that the predominant species of β-MSH was the 18-amino-acid form predicted by the location of dibasic cleavage sites. Furthermore, we found that it was present at a roughly equimolar ratio to d-α-MSH, consistent with it being a true neuropeptide.

Several lines of evidence suggest that the importance of β-MSH in regulating energy homeostasis in the human brain has not been fully appreciated. First, it is sufficient to reduce food intake in rodents [7], [73] with similar potency to α-MSH [18]. Second, human mutations affecting either a dibasic cleavage site [16] or a conserved tyrosine residue of β-MSH important for MC4R binding [17], [18] are strongly associated with obesity, suggesting that β-MSH is of direct clinical relevance and that its loss cannot be compensated by either α-MSH and/or d-α-MSH. Finally, dogs carrying frameshift mutations in POMC that affect β-MSH but not α-MSH lead to increased appetite and body weight [19]. Since β-MSH is not produced in rodents, hPSC-derived hypothalamic neurons provide a novel opportunity for studying the generation and regulation of this important peptide.

In both hPSC-derived hypothalamic neurons and in the human hypothalamus, we found that acetylated α-MSH was present at substantially lower concentrations than d-α-MSH, β-MSH, or β-EP (1-31). In agreement with these results, d-α-MSH was previously shown to be more abundant than α-MSH in the rodent hypothalamus [30], [74], [75], [76], [77] and human brain [78]. It is unlikely that the acetyl group was lost from α-MSH during sample preparation, since we did not observe significant peptide fragmentation indicative of degradation and acetylation is a stable peptide modification. In addition, the acetylated α-MSH peptide standard was efficiently recovered during solid phase extraction (Fig. S3), and acetylated α-MSH is robustly detected from mouse plasma using very similar sample preparation methods [66] (Fig. S4). It has been suggested that d-α-MSH may be acetylated immediately before secretion [9] and that leptin increases N-terminal acetylation of d-α-MSH [30], but concentrations of secreted α-MSH were too low in our culture system to enable these hypotheses to be directly tested. Further development of peptide quantification methods may allow these questions to be addressed in future studies.

Since both α-MSH and d-α-MSH are likely secreted, what might the roles of each of these peptides might be in human energy homeostasis? Previous studies using acute peptide injections suggested that d-α-MSH is less potent at reducing food intake in rodents than α-MSH [7], [65]. These findings have recently been challenged by a longer-term study in which both α-MSH and d-α-MSH were genetically deleted from mice, and then individually infused for 14 days into the mouse brain over a range of concentrations. This study revealed that both peptides could similarly reduce appetite and body weight in male mice [6], and indeed d-α-MSH may be a more potent agonist of MC4R than α-MSH [9]. We therefore suggest that, in humans, the relative importance of both d-α-MSH and β-MSH may have been underestimated, and that the assumption that acetylated α-MSH is the principal anorexigenic melanocortin peptide in humans should be reconsidered.

In our studies, β-EP (1-31) was present at 2–4 fold higher concentrations than d-α-MSH or β-MSH in hPSC-derived neurons (Figure 4B), in the human brain (Figure 4C), and upon induced secretion (Figure 6B). Control experiments suggest that this observation was likely not due to technical artefacts such as the degradation of standard peptides. It is possible that β-EP (1-31) is intrinsically more stable within the cell or more readily extracted by the methods we used. However, it is also noteworthy that it only requires a single cleavage event at Lys219-Arg220 (position 235-236 from translational start) to be liberated from larger precursor peptides, whereas the generation of either d-α-MSH or β-MSH requires at least two cleavage events. It is also possible that POMC cleavage is less efficient at the dibasic residues flanking α-MSH and β-MSH sequences than at Lys219-Arg220 due to steric or other reasons.

In addition to the well-characterised POMC-peptides α-MSH, d-α-MSH, β-MSH and β-EP, we also identified γ_1_-MSH [62] in one hPSC-derived hypothalamic neuron sample. Like α-MSH, d-α-MSH and β-MSH, the γ-MSH peptide is anorexigenic [79] but in contrast to the other MSH peptides it has higher affinity for MC3R than MC4R [80], [81]. Since γ-MSH was not consistently detected, it may be present at low concentrations, possibly due to O-glycosylation at the asparagine residue adjacent to its C-terminal dibasic cleavage site, which could sterically interfere with peptide cleavage [82], [83], [84], [85].

Predictions made by studying hypothalamic neurons derived from three distinct hPSC lines were largely confirmed in primary human hypothalamic samples. However, the human brain contained a larger repertoire of neuropeptides than we observed *in vitro*. Some of these differences might be explained by the distinct anatomical origin of neuropeptide-producing neurons, since our *in vitro* model generates cells with a predominantly ventral hypothalamic regional identity [31], whereas neurons producing gonadotropin releasing hormone (GNRH) are generated in the olfactory placode and then migrate into the hypothalamus [86], and neurons expressing glucagon like peptide 1 (GLP-1) project to the hypothalamus from nucleus of the solitary tract (NTS) of the brainstem [87]. Other differences could be explained by the maturation state of hPSC-derived hypothalamic neurons, which might express some neuropeptides at levels below the detection limit of the automated peptide identification pipeline we employed. The use of more sensitive LC-MS/MS instruments or the analysis of raw peptidomic data using new discovery tools [88] may reveal further biologically important peptides.

We found that POMC-derived peptides were robustly secreted upon depolarisation of hPSC-derived hypothalamic neurons and that the relative concentrations of β-MSH and β-EP were consistent with what we observed in cell pellets (Figures 4B and 6B). However, the relative ratio of secreted of d-α-MSH was unexpectedly low, less than half that of β-MSH. This could be due to the instability of d-α-MSH in the supernatant (though we did not detect many degradation products). Alternatively, these data could suggest the differential vesicle packaging and/or secretion efficiency of different POMC-derived peptides, which has been previously reported in mice *in vivo* [89]. The preferential release of POMC peptides with different physiological functions could be an important mechanism for regulating feeding behaviour in response to different physiological states.

Importantly, we found that exposure of hPSC-derived hypothalamic neurons to leptin significantly increased the concentration of d-α-MSH, β-MSH and β-EP (Figure 7C). The mechanism for leptin elevating POMC-derived peptide concentrations could be due to its previously observed effects on inducing POMC transcription and translation [26], [28], its effects on promoting prohormone processing by increasing PC1/3 and PC2 expression [29], or some combination of the two. Conversely, treatment of cultures with a broad-spectrum PC inhibitor that is likely to inhibit many PC enzymes at the concentration used [90], [91] significantly reduced concentrations of POMC-derived peptides. Together, these findings are consistent with observations made in animals, suggesting that this human cellular model system may have predictive power for understanding human-specific aspects of POMC biology, such as the regulation of β-MSH processing and secretion. The genetic or environmental manipulation of hPSC-derived hypothalamic neurons coupled with quantitative LC-MS/MS-based analysis may therefore provide insights into the molecular mechanisms regulating neuronal responsiveness and neuropeptide processing. The human-specific nature of several key findings in this study highlight the need for further human cellular studies into the central regulation of energy homeostasis, which has the potential to identify novel therapeutic targets for obesity.

## 5 Conclusion

Quantitative LC-MS/MS of both primary and stem cell-derived human hypothalamic neurons revealed that d-α-MSH and β-MSH are by far the most abundant melanocortins, suggesting that the widely-held assumption that α-MSH is the most important POMC-derived peptide for regulating energy homeostasis should be revisited. The production of human d-α-MSH, β-MSH and β-EP peptides is dynamically regulated by leptin, establishing the utility of stem cell-derived hypothalamic neurons for studying both POMC processing and neuronal responsiveness to exogenous factors.

## Funding

This work was supported by the Academy of Medical Sciences (SBF001∖1016), Medical Research Council (MR/P501967/1, MR/M009041/1, and Metabolic Diseases Unit, award 4050281695, MC UU 12012/3, MC UU 12012/5), Wellcome Trust (106262/Z/14/Z, 106263/Z/14/Z, PSAG/097), NIHR Cambridge Biomedical Research Centre, Prometeo Network (PROMETEOII/2014/075), Open Targets (OTAR0039), Red de Terapia Celular TerCel of the Instituto de Salud Carlos III, Spain (RD16/0011/0026), Mawer-Fitzgerald Endowment, Manchester Academic Health Sciences Centre, and by a project grant from MedImmune.

## Author contributions

PK and FTM conceived of the project, analysed data, and wrote the manuscript with input from all co-authors. PK performed hypothalamic differentiation with the help of MJ, JJ and BB, performed experiments, and prepared samples for LC-MS/MS. RK extracted and ran samples for LC-MS/MS and assisted with data analysis. PL helped develop methods for peptide extraction and assisted with sample preparation. VHP generated and helped interpret electron microscopic data. JP helped develop the quantitative LC-MS/MS SRM methodology and analysed human samples. AW contributed anti-POMC antibodies and the POMC ELISA protocol (performed by TB) and provided intellectual guidance together with FG, FR, ISF, and SOR, who also helped financially support this study.
