# Supplementary material for: Quantitative mass spectrometry for human melanocortin peptides in vitro and in vivo suggests prominent roles for β-MSH and desacetyl α-MSH in energy homeostasis
Source: Mol Metab. 2018 Aug 21;17:82–97. doi: 10.1016/j.molmet.2018.08.006 (PMC6197775; doi:10.1016/j.molmet.2018.08.006)
Supplement: Multimedia component 1 [file mmc1.docx]

**
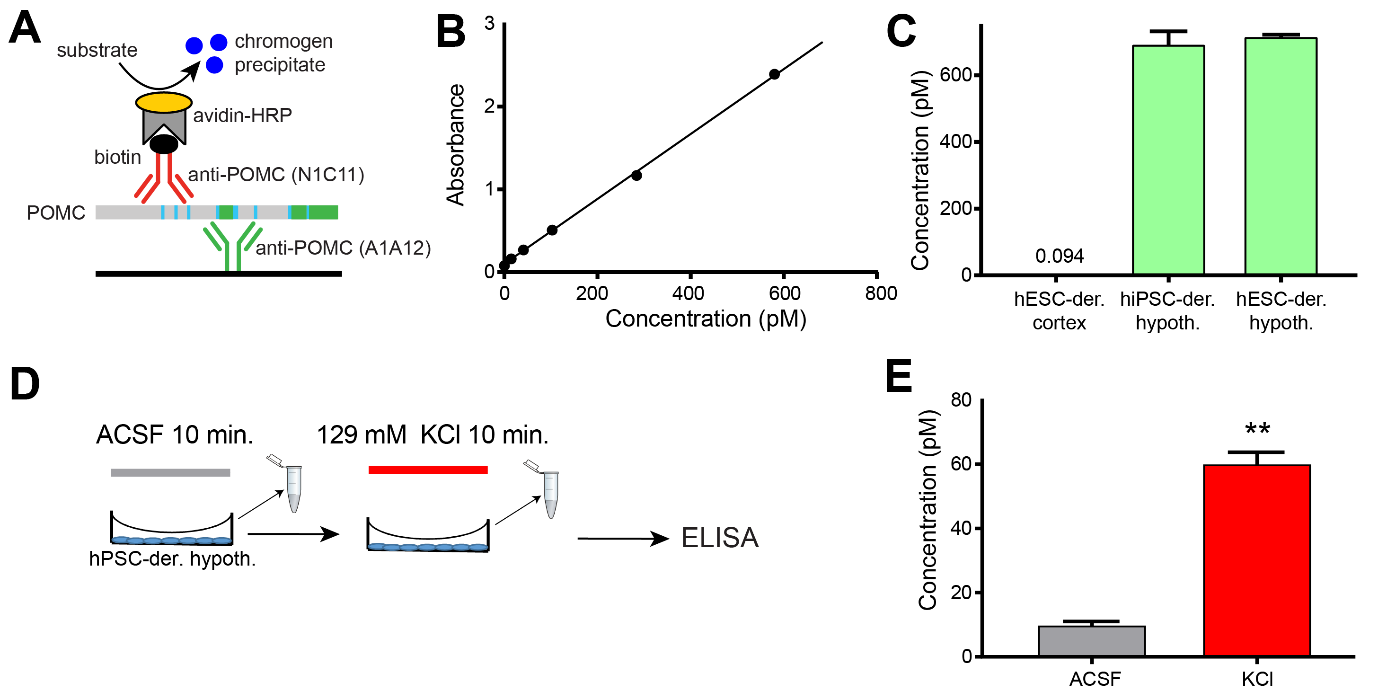
**

**Figure S1: Secretion of POMC in hPSC-derived hypothalamic neurons in the presence or absence of experimental depolarisation.**

**A)** Schematic diagram of the sandwich ELISA assay for the absorbance-based quantitative detection of full-length POMC and pro-ACTH. **B**) The ELISA standard curve generated using purified human POMC protein is linear over a broad range and enables quantification down to approximately 7.5 pM. **C**) POMC and/or pro-ACTH quantification from the supernatant of hPSCs differentiated to cortical neurons or hESC and hiPSC-derived hypothalamic neurons. N=2 independent cell lines, 3 replicate wells per cell line. **D**) Schematic diagram of stimulation experiments to test for depolarisation-induced increases in POMC and/or pro-ACTH secretion by ELISA. **E**) KCl-induced depolarisation increased measured concentrations of secreted POMC and/or pro-ACTH. N=1 experiment, 3 replicate wells. ACSF, artificial cerebrospinal fluid; der., derived; HRP, horseradish peroxidase; hypoth., hypothalamic; KCl, potassium chloride; **, P <0.01. Error bars show SEM.

**
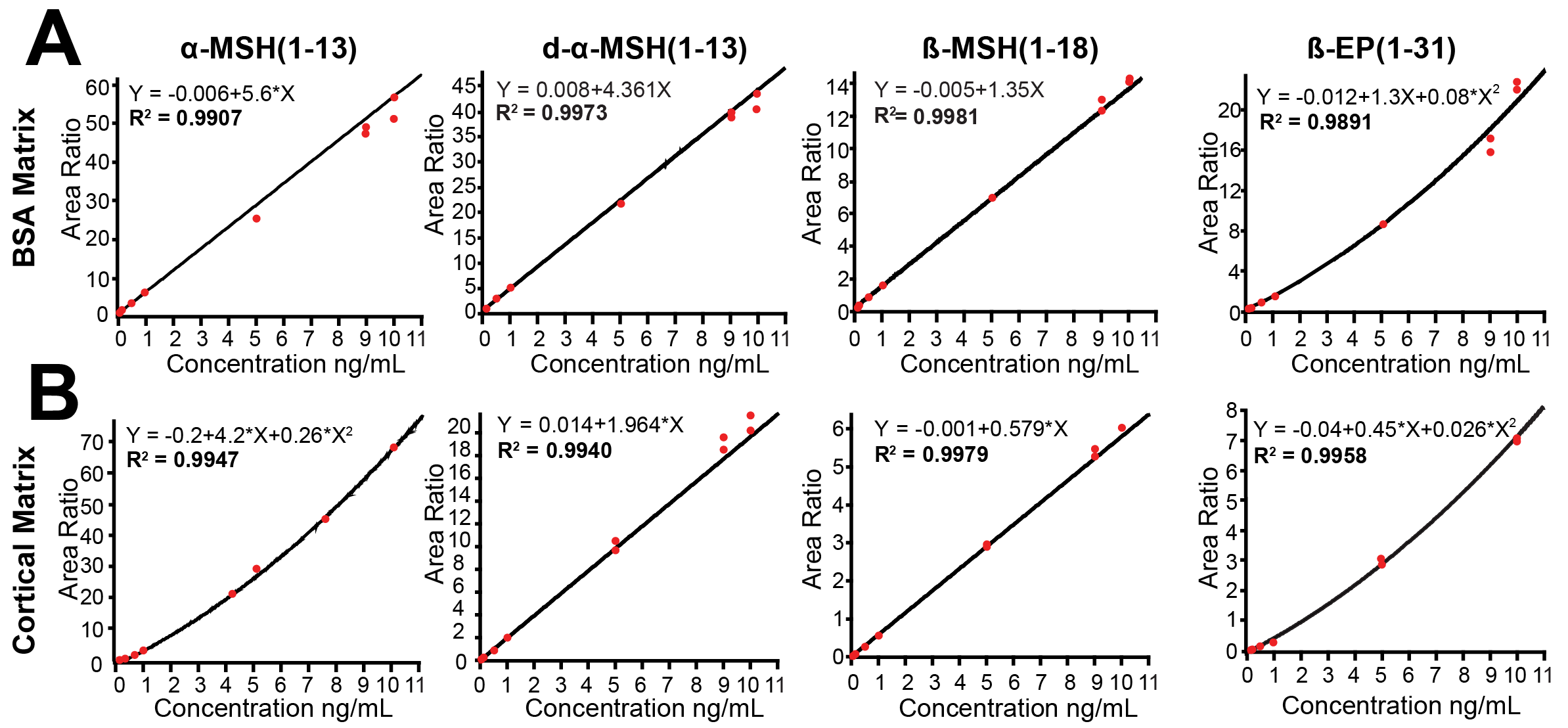
**

**Figure S2: Peptide quantification.**

Standard curves generated with known quantities of synthetic POMC-derived peptides enable accurate peptide quantification (R^2^>0.98) over a broad concentration range. Standards were added to either a BSA matrix (A) or a hPSC-derived cortical cell matrix (B).


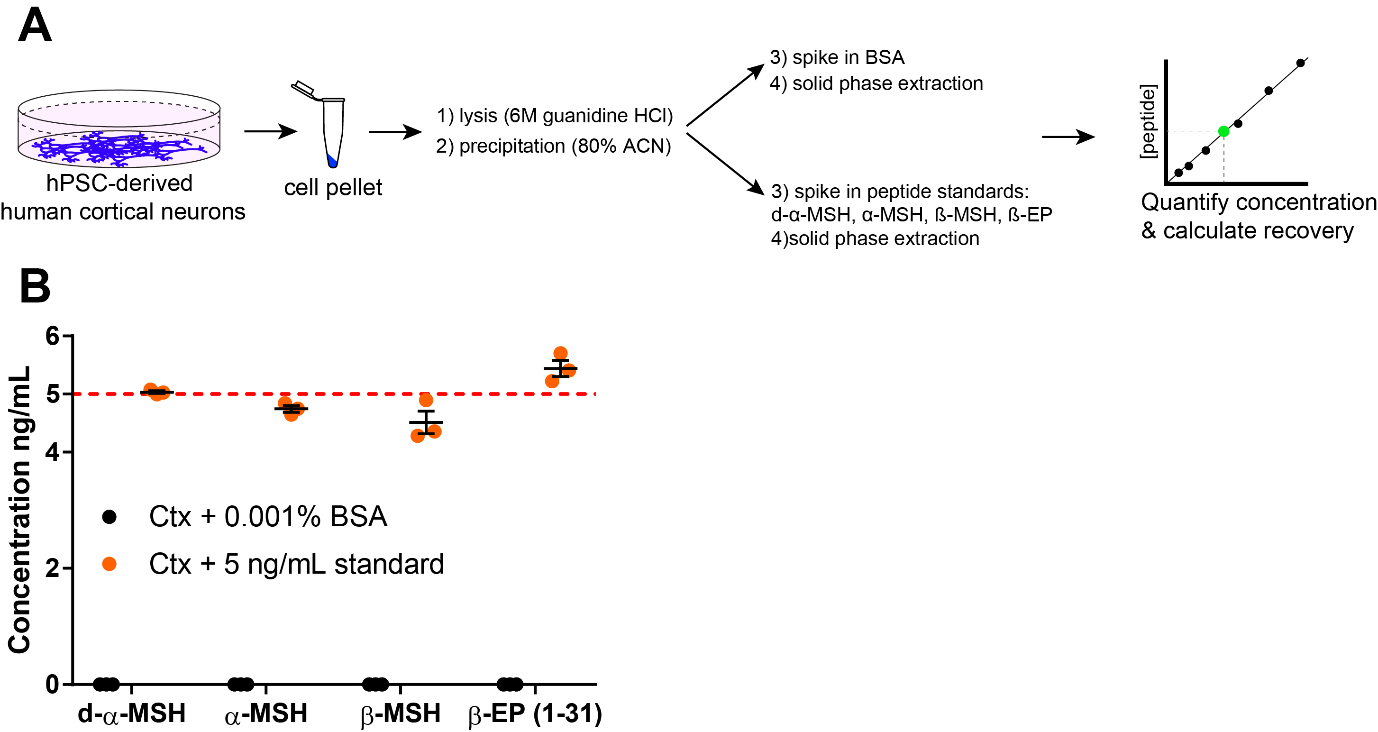


**Figure S3: Efficient recovery of POMC-derived standards after solid phase extraction.**

**A)** Peptides were extracted from hPSC-derived cortical neurons and then spiked with 0.001% BSA or 5 ng/mL of POMC-derived peptide standards, followed by solid phase extraction. Peptide concentrations were then quantified by LC-MS/MS. **B)** Concentrations measured in samples spiked with d-α-MSH(1-13), α-MSH(1-13), β-MSH(1-18) and β-EP(1-31) standards (orange circles) were close to the expected concentration of 5 ng/mL (dotted red line). Ctx, hPSC-derived cortical cell matrix.

**
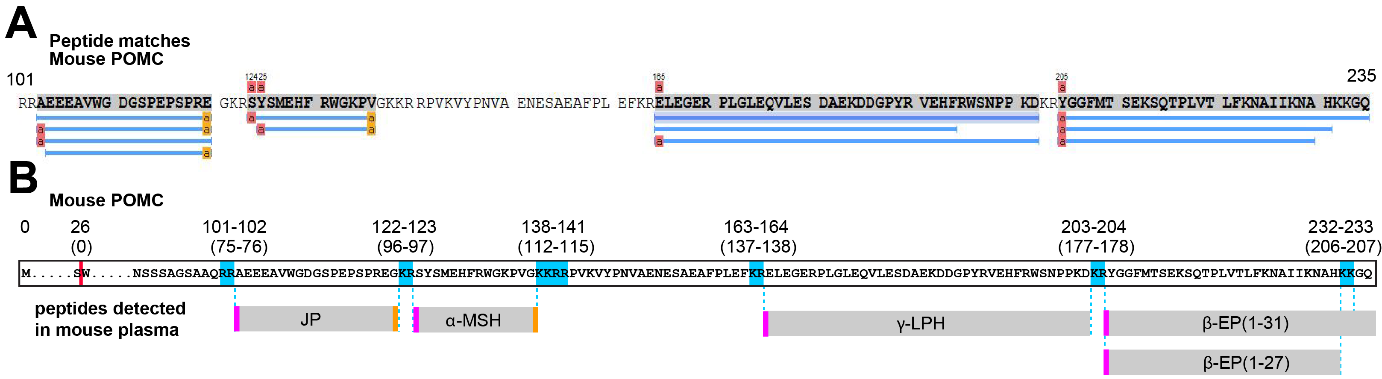
**

**Figure S4: Detection of acetylated α-MSH peptide in mouse plasma.**

**A)** Peptide alignments (blue bars) from nano-flow LC-MS/MS analysis of mouse plasma reveals N-terminally acetylated (red) and C-terminally amidated (orange) residues, including abundant acetylated and amidated α-MSH. **B)** Schematic diagram of alignments from (A), where dibasic cleavage residues are represented in blue, acetylated residues in magenta, and amidated residues in orange.

**
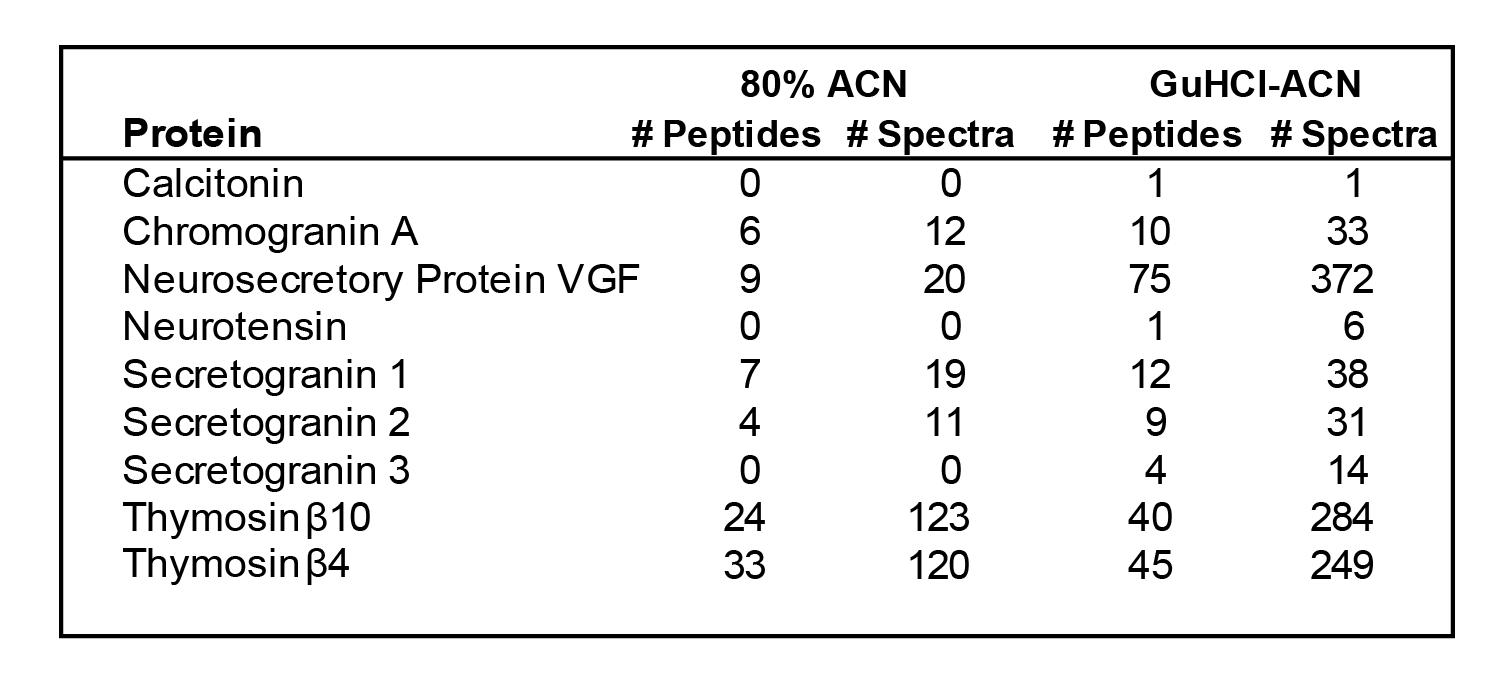
**

**Table S1: Comparison extraction methods for neuropeptides.**

Peptide extraction by either 80% ACN alone or 6M GuHCl followed by ACN yielded similar types of peptides detected by LC-MS/MS, but GuHCl-ACN extraction was associated with a larger number of peptides and a larger number of spectra supporting each peptide call.

**
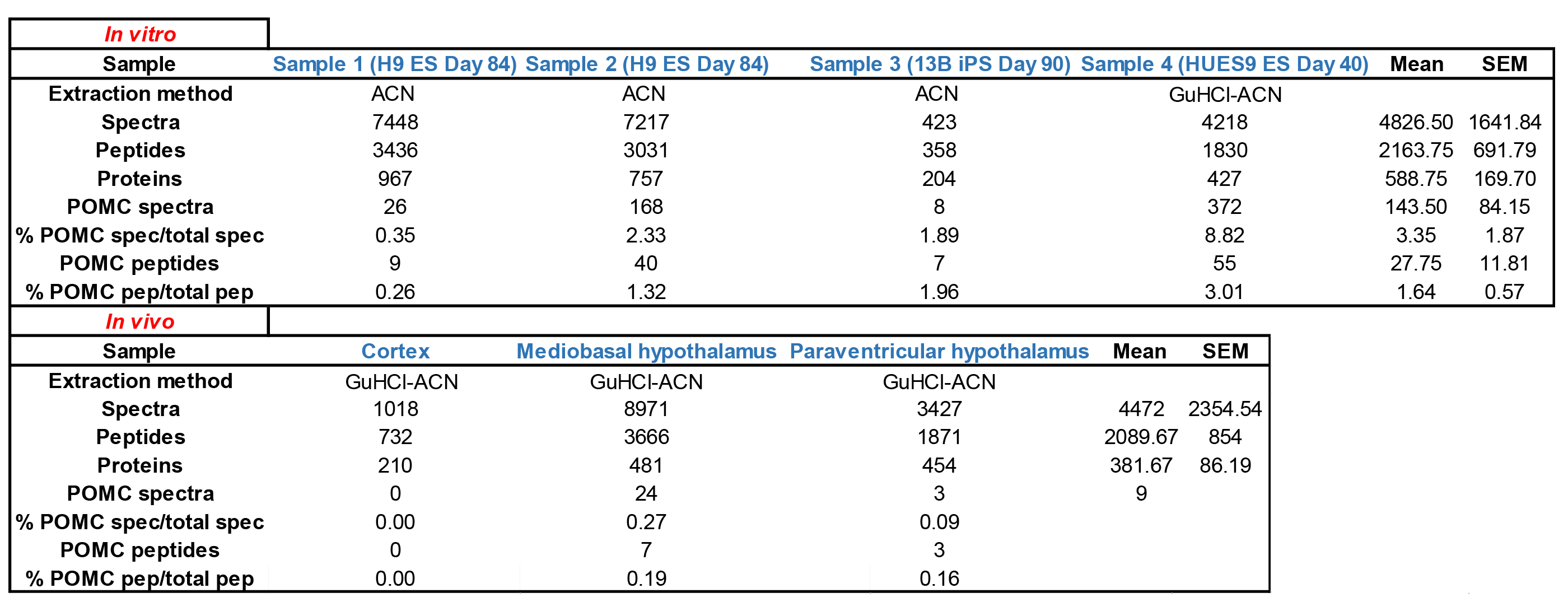
**

**Table S2: Quantitative summary of peptides detected *in vitro* and *in vivo.***

Quantitative summary of the number of spectra, peptides and unique proteins in hPSC-derived hypothalamic neurons (*in vitro*) as well as from primary human brain (*in vivo*). A summary of the number of POMC-derived peptides and supporting spectra is also shown, as well as the percentage for POMC spectra and peptides as a function of total peptides observed in each experiment.
